# Association between personality traits and medication adherence in people who live with type 2 diabetes and hypertension: A systematic review

**DOI:** 10.1192/j.eurpsy.2025.849

**Published:** 2025-08-26

**Authors:** K. Dimou, T. Stamoulis, M. Kourakos, E. Dragioti, S. Mantzoukas, M. Gouva

**Affiliations:** 1 Research Laboratory Psychology of Patients, Families and Health Professionals, Department of Nursing, School of Health Sciences, University of Ioannina; 2 Research Laboratory of Integrated Health, Care and Well-being, Department of Nursing. School of Health Sciences, University of Ioannina, Ioannina, Greece

## Abstract

**Introduction:**

Chronic diseases, such as type 2 diabetes and hypertension, are rapidly increasing worldwide. These conditions can have significant negative impacts on patients’ daily lives.

**Objectives:**

Our aim was to investigate the correlation between personality traits and medication adherence as part of self-care among individuals living with type 2 diabetes and hypertension.

**Methods:**

We conducted a thorough and comprehensive literature search for published articles in PsycINFO, CINAHL, and PubMed from inception to September 2024, following PRISMA guidelines. The included studies were primary quantitative research conducted in English and focused on adults aged 18 and above, diagnosed with hypertension or type 2 diabetes mellitus.

**Results:**

We identified a total of 41 studies that met our inclusion criteria, analyzing the relationship between personality traits and medication adherence. Specifically, 23 studies indicated that traits such as agreeableness were associated with improved medication adherence in individuals with type 2 diabetes, while traits such as extraversion and neuroticism were linked to reduced adherence. With regard to hypertension, 18 studies showed neuroticism was associated with decreased medication adherence, while extraversion was associated with increased medication adherence.

**Conclusions:**

Personality traits play a crucial role in self-care, with one of its key aspects being adherence to medication in chronic conditions such as type 2 diabetes and hypertension.

**Disclosure of Interest:**

None Declared

